# Ethyl pyruvate attenuates ventilation‐induced diaphragm dysfunction through high‐mobility group box‐1 in a murine endotoxaemia model

**DOI:** 10.1111/jcmm.14478

**Published:** 2019-06-10

**Authors:** Yung‐Yang Liu, Ning‐Hung Chen, Chih‐Hao Chang, Shih‐Wei Lin, Kuo‐Chin Kao, Han‐Chung Hu, Gwo‐Jyh Chang, Li‐Fu Li

**Affiliations:** ^1^ Chest Department Taipei Veterans General Hospital Taipei Taiwan; ^2^ Institutes of Clinical Medicine School of Medicine National Yang‐Ming University Taipei Taiwan; ^3^ Department of Internal Medicine, Division of Pulmonary and Critical Care Medicine Chang Gung Memorial Hospital Taoyuan Taiwan; ^4^ Department of Internal Medicine Chang Gung University Taoyuan Taiwan; ^5^ Department of Respiratory Therapy Chang Gung Memorial Hospital Taoyuan Taiwan; ^6^ Department of Respiratory Care, College of Medicine Chang Gung University Taoyuan Taiwan; ^7^ Graduate Institute of Clinical Medical Sciences Chang Gung University Taoyuan Taiwan

**Keywords:** endotoxaemia, ethyl pyruvate, high‐mobility group box‐1, mitochondria, ventilator‐induced diaphragm dysfunction

## Abstract

Mechanical ventilation (MV) can save the lives of patients with sepsis. However, MV in both animal and human studies has resulted in ventilator‐induced diaphragm dysfunction (VIDD). Sepsis may promote skeletal muscle atrophy in critically ill patients. Elevated high‐mobility group box‐1 (HMGB1) levels are associated with patients requiring long‐term MV. Ethyl pyruvate (EP) has been demonstrated to lengthen survival in patients with severe sepsis. We hypothesized that the administration of HMGB1 inhibitor EP or anti‐HMGB1 antibody could attenuate sepsis‐exacerbated VIDD by repressing HMGB1 signalling. Male C57BL/6 mice with or without endotoxaemia were exposed to MV (10 mL/kg) for 8 hours after administrating either 100 mg/kg of EP or 100 mg/kg of anti‐HMGB1 antibody. Mice exposed to MV with endotoxaemia experienced augmented VIDD, as indicated by elevated proteolytic, apoptotic and autophagic parameters. Additionally, disarrayed myofibrils and disrupted mitochondrial ultrastructures, as well as increased HMGB1 mRNA and protein expression, and plasminogen activator inhibitor‐1 protein, oxidative stress, autophagosomes and myonuclear apoptosis were also observed. However, MV suppressed mitochondrial cytochrome C and diaphragm contractility in mice with endotoxaemia (*P < *0.05). These deleterious effects were alleviated by pharmacologic inhibition with EP or anti‐HMGB1 antibody (*P < *0.05). Our data suggest that EP attenuates endotoxin‐enhanced VIDD by inhibiting HMGB1 signalling pathway.

## 1 INTRODUCTION

Mechanical ventilation (MV) is a life‐saving treatment for patients suffering from acute respiratory failure, but it may also lead to patient's reliance on ventilators because of a resultant and rapid decline of diaphragm muscle endurance and strength, which is termed ventilator‐induced diaphragmatic dysfunction (VIDD).^1^, ^2^, ^3^ Numerous studies have determined that VIDD could raise weaning failure rate, intensive care unit (ICU) stay and medical expenditures.^2^, ^3^ However, the mechanisms of VIDD, presumably encompassing a multistep process that involves oxidative loads, muscle atrophy (arising from calpain, caspase‐3, the autophagy‐lysosomal pathway [ALP] and ubiquitin‐proteasome system [UPS] activation), structural damage and muscle fibre remodelling, have not been fully explored.^4^, ^5^, ^6^ Therefore, a detailed understanding of the precise mechanisms underlying VIDD is critical for the development of potential strategies to reduce the prolonged use of MV and thus ICU mortality.

Sepsis is a major risk factor for ICU patients developing diaphragm dysfunction.^2^, ^3^, ^7^ During the progress of sepsis, deleterious host response to infectious constituents, such as lipopolysaccharide (LPS), may induce an inflammatory cascade and cause ventilator‐associated pneumonia (VAP), subsequently leading to multiple organ failure.^5^, ^8^, ^9^, ^10^ Animal studies have demonstrated that infection is a principal cause of abnormal diaphragm activities.^8^, ^11^ Sepsis‐mediated diaphragmatic weakness and VIDD involve common molecular pathways, namely excessive oxidative loads and mitochondrial abnormalities within the injured diaphragm myofibrils, suggesting that sepsis may be an accessory contributor to VIDD.^5^, ^6^, ^8^, ^10^ In an acute lung injury (ALI), reactive oxygen species (ROS) are the major oxidants in the diaphragm and can be produced in mitochondria, sarcoplasmic reticula, sarcolemma, transverse tubes and cytosol within 6 hours of MV.^12^, ^13^ Furthermore, sepsis and MV‐induced oxidative stress may deteriorate diaphragm contractility and are critical contributors to proteolytic pathway activation.^14^, ^15^, ^16^ The primary proteases in the skeletal muscle consist of (1) UPS, (2) calcium‐related proteases and (3) lysosomal enzymes.^10^, ^17^ The up‐regulation of muscle‐specific ubiquitin E3 ligases F‐box protein atrogin‐1 and muscle RING‐finger proteins‐1 (MuRF‐1) is crucial for the proteolysis of monomeric myofibrillar proteins in the diaphragms of animals and patients using MV.^16^, ^17^, ^18^ Increased autophagosome formation also occurs in MV‐augmented diaphragmatic weakness, as reflected in an elevation of autophagic biomarker microtubule‐related protein light chain (LC) 3.^19^, ^20^, ^21^ Mitochondria are a principal source of diaphragmatic ROS and act as a pivotal upstream regulator that induces the molecular pathways engendering diaphragm muscle atrophy during endotoxaemia or MV.^22^, ^23^ In addition, myonuclear apoptosis can also be accelerated by mitochondrial ROS, elevated cellular calcium levels and sarcoplasmic reticulum stress‐induced activation of calpain and caspase‐3.^20^, ^24^ Furthermore, sepsis and MV‐induced oxidative stress may up‐regulate the production of inflammatory mediators, including high‐mobility group box 1 (HMGB1), interleukin 6 (IL‐6) and plasminogen activator inhibitor‐1 (PAI‐1),^9^, ^15^, ^25^, ^26^, ^27^, ^28^, ^29^ and subsequently impair diaphragm activities.

Rodent studies of endotoxaemia have revealed that toll‐like receptor 4 (TLR4) modulates diaphragm injury by activating the nuclear factor‐κB (NF‐κB) pathway.^11^, ^30^ In our previous mouse study investigating sepsis, the reduced ALP and mitigated mitochondrial ultrastructural changes were observed by inhibiting TLR4/NF‐κB signalling through genetic manipulation of TLR4 using homozygous knockout.^31^ TLR4 is the most renowned receptor of the TLR family and is crucial for the recognition of the damage‐associated molecular pattern (DAMP), including extracellular matrix components, HMGB1 and LPS.^30^, ^32^ Although inhibition of TLR4 could be an effective therapeutic strategy for VIDD, in light of the potential risk of increased infectious complications using this approach, targeting endogenous TLR4 ligands such as HMGB1 could be more prudent. Related studies have demonstrated that the expression levels of HMGB1 are associated with diaphragmatic dysfunction in caecal ligation and puncture in animals and that the administration of anti‐HMGB1 antibodies can effectively attenuate sepsis‐induced diaphragm dysfunction in septic rats.^26^, ^28^ Several related studies have demonstrated that ethyl pyruvate, a potent free radical scavenger, can inhibit HMGB1 production and improve the survival of critically ill patients.^33^, ^34^ Nevertheless, the use of ethyl pyruvate for the management of VIDD is still unexplored.

Murine endotoxaemia models have been employed to recapitulate human sepsis for nearly a century.^35^ In this study, we investigated that the impact of MV with or without LPS and related HMGB1 signalling contributes to VIDD using a murine model of endotoxaemia. We hypothesized that the administration of ethyl pyruvate or anti‐HMGB1 antibody would diminish HMGB1 expression, diaphragmatic structural damage, generation of free radicals, proteolytic protein synthesis and mitochondrial dysfunction and restore muscle contractility in the diaphragm of mice with or without endotoxaemia exposed to MV.

## 2 MATERIALS AND METHODS

### 2.1 Experimental animals

Male C57BL/6 mice, weighing between 20 and 25 g, aged between 6 and 8 weeks, were obtained from the National Laboratory Animal Center (Taipei, Taiwan). The experiments were conducted in strict accordance with the National Institutes of Health Guidelines on the Use of Laboratory Animals. The Institutional Animal Care and Use Committee of Chang Gung Memorial Hospital approved the protocol (Permit number: 2015101201). All surgery was performed under zoletil and xylazine anaesthesia, and all efforts were made to minimize suffering.

### 2.2 Experimental groups

Animals were randomly distributed into six groups in each experiment: group 1, non‐ventilated control wild‐type mice with normal saline; group 2, non‐ventilated control wild‐type mice with LPS; group 3, tidal volume (V_T_) 10 mL/kg wild‐type mice with normal saline; group 4, V_T_ 10 mL/kg wild‐type mice with LPS; group 5, V_T_ 10 mL/kg wild‐type mice after ethyl pyruvate (100 mg/kg) administration with LPS; group 6, V_T_ 10 mL/kg wild‐type mice after anti‐HMGB1 antibody (100 mg/kg) with LPS; group 7, V_T_ 10 mL/kg wild‐type mice after ethyl pyruvate (50 mg/kg) administration with LPS; group 8, V_T_ 10 mL/kg wild‐type mice after anti‐HMGB1 antibody (50 mg/kg) administration with LPS; group 9, V_T_ 10 mL/kg wild‐type mice after anti‐HMGB1 isotype control antibody (100 mg/kg) administration with LPS. In groups 1‐6, three mice underwent transmission electron microscopy (TEM) and specific force, and five mice underwent measurement for immunohistochemistry assay, inflammatory cytokines, protein carbonyl groups, superoxide dismutase, terminal deoxynucleotidyl transferase‐mediated dUTP‐biotin nick end‐labelling (TUNEL) assay, and Western blots. In groups 7‐9, five mice underwent measurement for HMGB1 and Western blots.

### 2.3 Ventilator protocol

We used our established murine model of VILI, as described previously.^9^, ^31^ Briefly, a 20‐gauge angiocatheter was inserted into the tracheotomy orifice of mice and general anaesthesia was maintained by regular intraperitoneal administration of zoletil 50 (5 mg/kg) and xylazine (5 mg/kg) at the beginning of the experiment and every 30 minutes. The depth of anaesthesia was monitored from heart rates, respiratory rates and limb reflexes induced by paw and tail pinches performed periodically. The mice were placed in a supine position on a heating blanket and then attached to a Harvard apparatus ventilator, model 55‐7058 (Harvard Apparatus, Holliston, MA), set as tidal volume of 10 mL/kg at a rate of 100 breaths per min, for 8 hours while breathing room air with zero end‐expiratory pressure. Schellekens et al and our previous work demonstrated that 8‐hour MV in mice can induce increased cytokine expression, lysosomal autophagy and diaphragm atrophy.^9^, ^31^ At the end of the study, heparinized blood was taken from the arterial line for analysis of arterial blood gas, and the mice were sacrificed. The non‐ventilated control mice were anaesthetized and sacrificed immediately.

### 2.4 LPS and pharmacological inhibitors

Mice were administrated intravenously with either 1 mg/kg of Salmonella typhosa LPS (Lot 81H4018; Sigma Chemical Co., St. Louis, MO) or an equivalent volume of normal saline via the internal jugular vein as a control. After 1 hour of spontaneous respiration to allow for developing a septic response, the mice were subjected to MV for 8 hours.^9^, ^31^, ^36^ Two doses of ethyl pyruvate (Sigma, St Louis, MO) were administered intraperitoneally. The first dose was administered 30 minutes before the mice were subjected to MV and the second dose was used after the mice were subjected to 4 hours of MV.^29^, ^34^ Anti‐HMGB1 antibody 100 mg/kg (chicken anti‐pig HMGB1 polyclonal antibody, SHINO‐TEST, Tokyo, Japan) or isotype control antibody (non‐immune immunoglobulin G, SHINO‐TEST, Tokyo, Japan) was administered intravenously 30 minutes before the start of MV.^28^ The doses of ethyl pyruvate and anti‐HMGB1 were chosen on the basis of our and other studies that showed 100 mg/kg ethyl pyruvate or anti‐HMGB1 had better effects on inhibiting HMGB1 activity.^28^, ^29^, ^34^

### 2.5 Detection of cytokines in the bronchoalveolar lavage fluid

PAI‐1, with a lower detection limit of 0.02 ng/mL, and HMGB1 (1 ng/mL) were detected in bronchoalveolar lavage (BAL) fluid using immunoassay kits containing primary polyclonal anti‐mouse antibodies that were cross‐reactive with rat and mouse PAI‐1 and HMGB1 (PAI‐1: Molecular Innovations, Inc, Southfield, MI; HMGB1: Shino‐Test corporation, Kanagawa, Japan). Each sample was run in duplicate, according to the manufacturer's instructions.

### 2.6 Immunoblot analysis

The diaphragms were homogenized in 1 mL of lysis buffer (20 mmol/L HEPES pH 7.4), 1% Triton X‐100, 10% glycerol, 2 mmol/L ethylene glycol‐bis (β‐aminoethyl ether)‐N, N, N′, N′‐tetraacetic acid, 50 μmol/L β‐glycerophosphate, 1 mmol/L sodium orthovanadate, 1 mmol/L dithiotreitol, 400 μmol/L aprotinin and 400 μmol/L phenylmethylsulphonyl fluoride), transferred to Eppendorf tubes and placed on ice for 15 minutes. Tubes were centrifuged at 15 350*g* for 10 minutes at 4°C and supernatant was flash‐frozen. The total protein concentration was detected by Bradford protein assay kit (Thermo Fisher Scientific Inc, Waltham, MA). Crude cell lysates associated with the total protein content were matched for protein concentration (30 μg per well for caspase‐3 and LC3‐II; 60 μg per well for calpain, atrogin‐1 and MuRF‐1), resolved on a 10% bis‐acrylamide gel and electrotransferred to Immobilon‐P membranes (Millipore Corp., Bedford, MA). For the assay of calpain, caspase‐3, atrogin‐1, MuRF‐1, LC3‐II and glyceraldehydes‐phosphate dehydrogenase, Western blot analyses were analysed with respective antibodies (New England BioLabs, Beverly, MA and Santa Cruz Biotechnology, Santa Cruz, CA). Blots were developed by enhanced chemiluminescence (NEN Life Science Products, Boston, MA).

### 2.7 Immunohistochemistry

The diaphragms were paraffin embedded, sliced at 4 μm, deparaffinized, antigen unmasked in 10 mmol/L sodium citrate (pH 6.0), incubated with rabbit HMGB1 primary antibody (1:100; Santa Cruz Biotechnology, Santa Cruz, CA) and biotinylated goat anti‐rabbit secondary antibody (1:100) according to the manufacturer's instruction for an immunohistochemical kit (Santa Cruz Biotechnology, Santa Cruz, CA). The specimens were further conjugated with horseradish peroxidase‐streptoavidin complex, detected with a diaminobenzidine (DAB) substrate mixture and counterstained by haematoxylin. A dark‐brown DAB signal, identified by arrows, indicated positive staining of HMGB1 of muscle fibres, whereas shades of light blue signified non‐reactive cells.

### 2.8 Real‐time polymerase chain reaction

For isolating total RNA, the lung tissues were homogenized in TRIzol reagents (Invitrogen Corporation, Carlsbad, CA), according to the manufacturer's instructions. Total RNA (1 μg) was reverse transcribed using a GeneAmp polymerase chain reaction (PCR) system 9600 (PerkinElmer, Life Sciences, Inc, Boston, MA), as previously described.^31^ The following primers were used for real‐time polymerase chain reaction: HMGB1, forward primer 5′‐TGGCAAAGGCTGACAAGGCTC‐3′ and reverse primer 5′‐GGATGCTCGCCTTTGATTTTGG‐3′ and GAPDH as internal control, forward primer 5′‐GGAGCGAGACCCCACTAACA‐3′ and reverse primer 5′‐ACATACTCAGCACCGGCCTC‐3′ (Protech Technology Enterprise Co., Ltd., Taipei, Taiwan).^37^, ^38^ All quantitative PCR reactions using SYBR Master Mix were performed on a CFX96 Touch Real‐Time PCR Detection system (Bio‐Rad Laboratories, Inc, Hercules, CA). All PCR reactions were performed in duplicate and heated to 95°C for 5 minutes followed by 40 cycles of denaturation at 95°C for 10 seconds, and annealing at 55°C for 30 seconds. The relative gene expression was calculated using 2^−ΔΔCT^ method and the standard curves (cycle threshold values vs template concentration) were prepared for each target gene and for the internal control (GAPDH) in each sample. The specific gene's cycle threshold (Ct) values were normalized to the GAPDH and compared with the non‐ventilated control group with LPS that was assigned a value of 1 to calculate the relative fold change in expression.

### 2.9 Statistical evaluation

The Western blots were quantitated using a National Institutes of Health (NIH) image analyser Image J 1.27z (National Institutes of Health, Bethesda, MD) and presented as arbitrary units. Values were expressed as the mean ± SD from at least five separate experiments. The data of protein oxidation, superoxide dismutase, specific force, histopathologic assay and oxygenation were analysed using Statview 5.0 (Abascus Concepts, Cary, NC; SAS Institute). All results of real‐time PCR and Western blots were normalized to the non‐ventilated control wild‐type mice with LPS. ANOVA was used to assess the statistical significance of the differences, followed by multiple comparisons with a Scheffe′s test, and a *P* < 0.05 was considered statistically significant. We have performed the Shapiro‐Wilk normality test and verify that all data are parametric (*P* > 0.05). Additional details, including measurement of diaphragm force‐frequency relationships, immunoblot analysis, immunohistochemistry, mitochondrial isolation, measurement of diaphragmatic oxidative stress and antioxidant enzyme expression, TEM and TUNEL assay were performed as previously described.^9^, ^31^

## 3 RESULTS

### 3.1 Reduction of the effects of MV on endotoxin‐enhanced VIDD, oxygen radicals and inflammatory cytokines using ethyl pyruvate

**Figure 1:**
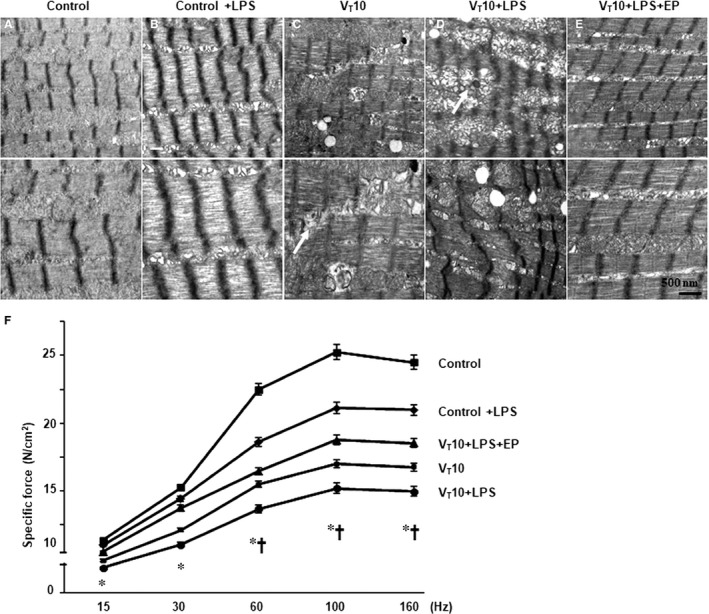
Electron microscopy and muscle force‐frequency activity of the diaphragm. Representative micrographs of the longitudinal sections of diaphragm (×20 000: upper panel; ×40 000: lower panel) were from the same diaphragms of non‐ventilated control mice and mice ventilated at a tidal volume (V_T_) of 10 mL/kg (V_T_ 10) for 8 h with or without LPS administration (n = 3 per group). (A and B) Non‐ventilated control wild‐type mice with or without LPS treatment: normal sarcomeres with distinct A bands, I bands and Z bands; (C) 10 mL/kg wild‐type mice without LPS treatment (normal saline): increase of diaphragmatic disarray; (D) 10 mL/kg wild‐type mice with LPS treatment: disruption of sarcomeric structure with loss of mitochondrial swelling, streaming of Z bands and collection of lipid droplets; (E) 10 mL/kg wild‐type mice pretreated with ethyl pyruvate: reduction of diaphragmatic disruption. (F) Diaphragm muscle‐specific force production was measured as described in Materials and Methods2. Mitochondrial swelling with concurrent loss of cristae and autophagosomes containing heterogeneous cargo are identified by arrows. Ethyl pyruvate, 100 mg/kg, was given intraperitoneally 30 min before mechanical ventilation and 4 h after mechanical ventilation. *P < 0.05 vs the non‐ventilated control mice with LPS treatment; ^†^ *P* < 0.05 vs all other groups. Scale bar represents 500 nm. EP, ethyl pyruvate; Hz, hertz; LPS, lipopolysaccharide; N, Newton

**Figure 2:**
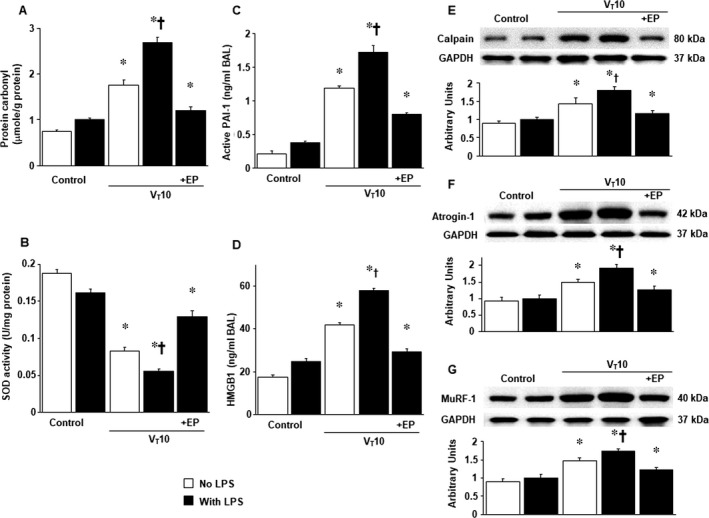
Ethyl pyruvate abrogated endotoxin‐augmented mechanical ventilation‐induced oxidative stress, inflammatory cytokines, calpain, atrogin‐1 and MuRF‐1 expression in the diaphragm. (A) protein carbonyl groups (diaphragm), (B) SOD (diaphragm), (C) BAL fluid active PAI‐1 and (D) BAL fluid HMGB1 were from the non‐ventilated control mice and mice ventilated at a tidal volume of 10 mL/kg for 8 h with or without LPS administration (n = 5 per group). Western blots were performed using antibodies that recognize calpain (E), atrogin‐1 (F), MuRF‐1 (G) and GAPDH expression from the diaphragms of non‐ventilated control mice and mice ventilated at a tidal volume of 10 mL/kg for 8 h with or without LPS administration (n = 5 per group). Arbitrary units were expressed as relative calpain, atrogin‐1 and MuRF‐1 activation (n = 5 per group). Ethyl pyruvate, 100 mg/kg, was given intraperitoneally 30 min before mechanical ventilation and 4 h after mechanical ventilation. *P < 0.05 vs the non‐ventilated control mice with LPS treatment; ^†^ *P* < 0.05 vs all other groups. BAL, bronchoalveolar lavage; GAPDH, glyceraldehydes‐phosphate dehydrogenase; HMGB1, high‐mobility group box‐1; MuRF‐1, muscle ring finger‐1; PAI‐1, plasminogen activator inhibitor‐1; SOD, sodium dismutase

We employed MV (10 mL/kg) at room temperature for 8 hours to elicit VIDD in mice. The physiological conditions at the beginning and end of MV are listed in Table S1. Normovolemic status was sustained in the mice by monitoring their mean arterial pressure. The dose‐dependent responses of ethyl pyruvate and anti‐HMGB1 used in this study were described in supplementary data (Figure S1). TEM was performed to explore MV‐ and LPS‐induced changes in the diaphragm ultrastructures. Compared with mice without endotoxaemia (normal saline only) subjected to V_T_ 10 mL/kg and the non‐ventilated controls, mice with endotoxaemia subjected to V_T_ 10 mL/kg exhibited higher disarrangements in diaphragmatic myofibrillar structures with larger lipid droplets, unclear A‐ and I‐bands, tortured Z‐bands and mitochondrial swelling (Figure 1A‐D). The administration of ethyl pyruvate substantially attenuated damage to the diaphragmatic fibres (Figure 1E). To determine the effects of sepsis and MV on diaphragm contractile conditions, we measured diaphragm muscle‐specific force generation. Decreased diaphragm contractilities were observed in mice with endotoxaemia subjected to MV compared with those without endotoxaemia subjected to MV and the non‐ventilated control mice (Figure 1F). The administration of ethyl pyruvate substantially suppressed MV‐ and endotoxin‐mediated increases in diaphragmatic weakness. Several studies have indicated the crucial roles of MV‐induced imbalances among oxidative loads, antioxidant capacity and inflammatory cytokines in worsening VIDD.^4^, ^5^, ^6^ Increased levels of active PAI‐1, HMGB1 and protein carbonyl groups and decreased production of superoxide dismutase were evident in mice with endotoxaemia subjected to MV compared to those without endotoxaemia subjected to V_T_ 10 mL/kg and the non‐ventilated control mice (Figure 2A‐D). However, a reversal of these features occurred after the administration of ethyl pyruvate.

### 3.2 Suppression of the effects of MV on endotoxin‐augmented calpain, atrogin‐1 and MuRF‐1 expression through the use of ethyl pyruvate

Western blot analyses were carried out to identify the effects of MV on the endotoxin‐augmented UPS related to VIDD. Total calpain, atrogin‐1 and MuRF‐1 levels were higher in mice with endotoxaemia subjected to V_T_ 10 mL/kg than in those without endotoxaemia subjected to V_T_ 10 mL/kg and the non‐ventilated control mice (Figure 2E‐G). Administering ethyl pyruvate substantially alleviated the elevated expression levels of calpain, atrogin‐1 and MuRF‐1 caused by endotoxaemia and 10 mL/kg MV.

### 3.3 Inhibition of the effects of MV on endotoxin‐exacerbated HMGB1 mRNA and HMGB1 protein expression through the use of ethyl pyruvate and anti‐HMGB1 antibody

**Figure 3:**
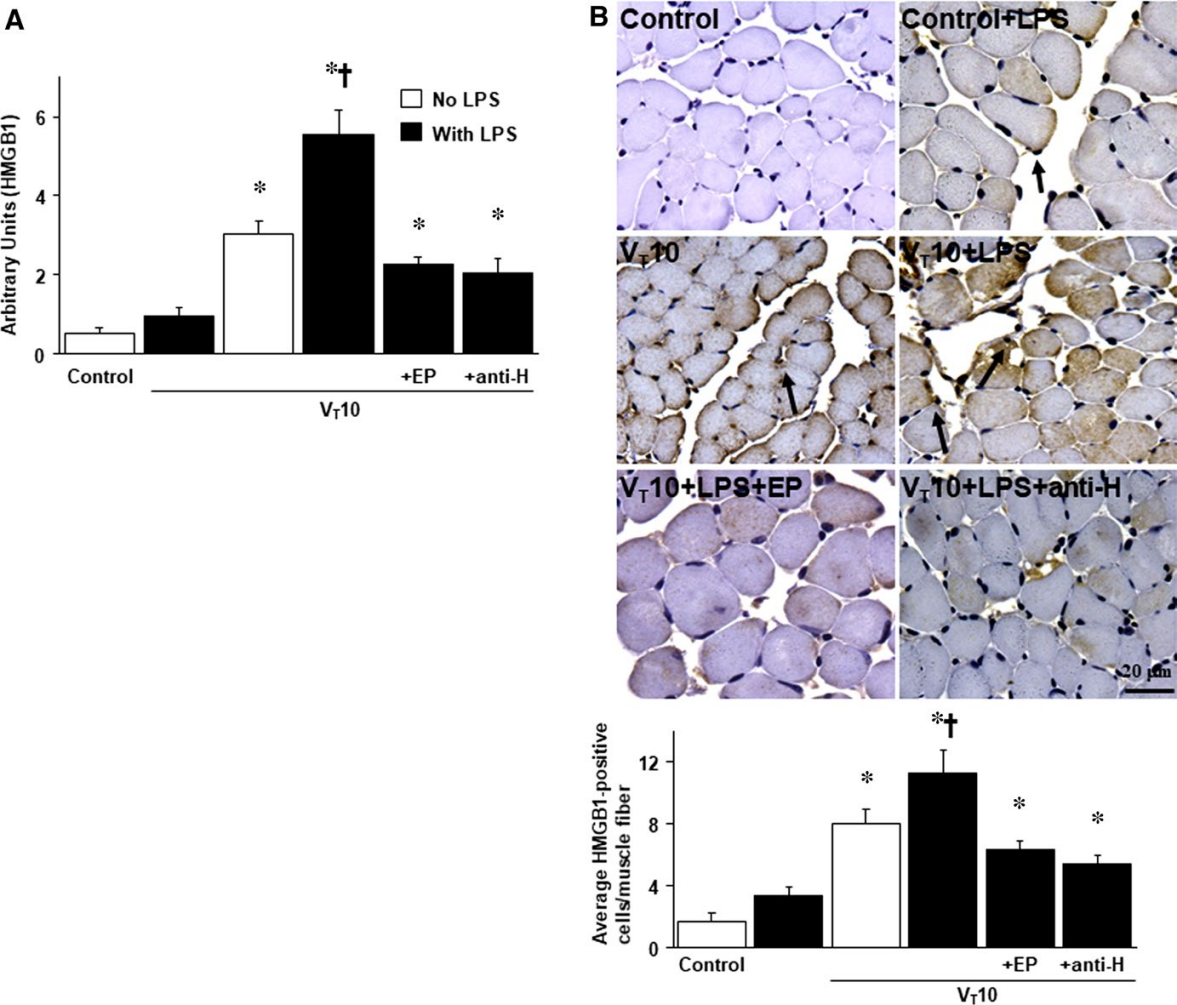
Ethyl pyruvate and anti‐HMGB1 antibody inhibited endotoxin‐aggravated mechanical ventilation‐enhanced HMGB1 mRNA activation and HMGB1 protein expression. (A) Real‐time PCR performed for HMGB1 mRNA expression was from the diaphragms of non‐ventilated control mice and mice ventilated at a tidal volume of 10 mL/kg for 8 h with or without LPS administration (n = 5 per group). Arbitrary units were expressed as the ratio of HMGB1 mRNA to GAPDH (n = 5 per group). (B) Representative micrographs (x400) with HMGB1 staining of paraffin diaphragm sections and quantification were from the diaphragms of non‐ventilated control mice and mice ventilated at a tidal volume of 10 mL/kg for 8 h with or without LPS administration (n = 5 per group). Ethyl pyruvate, 100 mg/kg, was given intraperitoneally 30 min before mechanical ventilation and 4 h after mechanical ventilation. Anti‐HMGB1 antibody, 100 mg/kg, was administered intravenously 30 min before the start of ventilation. A dark‐brown diaminobenzidine signal identified by arrows indicates positive staining for HMGB1 in the diaphragm, whereas shades of bluish tan signify non‐reactive cells. *P < 0.05 vs the non‐ventilated control mice with LPS treatment; ^†^ *P* < 0.05 vs all other groups. Scale bars represent 20 μm. Anti‐H, anti‐HMGB1 antibody; PCR, polymerase chain reaction

HMGB1 is an inflammatory cytokine and an intracellular regulator of transcription and its activation has been associated with diaphragm weakness in a sepsis model of rats.^15^, ^26^, ^39^ Real‐time PCR was performed to measure the effects of MV on endotoxin‐associated HMGB1 mRNA expression in the diaphragm (Figure 3A**)**. The expression level of HMGB1 mRNA was up‐regulated in mice with endotoxaemia subjected to V_T_ 10 mL/kg compared to those without endotoxaemia subjected to V_T_ 10 mL/kg and the non‐ventilated control mice. The increase in HMGB1 mRNA expression after MV was substantially reduced by the administration of either ethyl pyruvate or anti‐HMGB1 antibody (Figure 3A). Immunohistochemistry was employed to discern the effects of HMGB1 expression in endotoxin‐mediated VIDD (Figure 3B). A substantial elevation in the number of diaphragm muscle fibres positively stained for HMGB1 was observed in mice with endotoxaemia subjected to V_T_ 10 mL/kg compared to those without endotoxaemia subjected to V_T_ 10 mL/kg and the non‐ventilated control mice. The elevated HMGB1 expression levels after MV were substantially diminished after the administration of either ethyl pyruvate or anti‐HMGB1 antibody (Figure 3B).

### 3.4 Reduction of the effects of MV on endotoxin‐augmented VIDD by ethyl pyruvate and anti‐HMGB1 antibody

**Figure 4:**
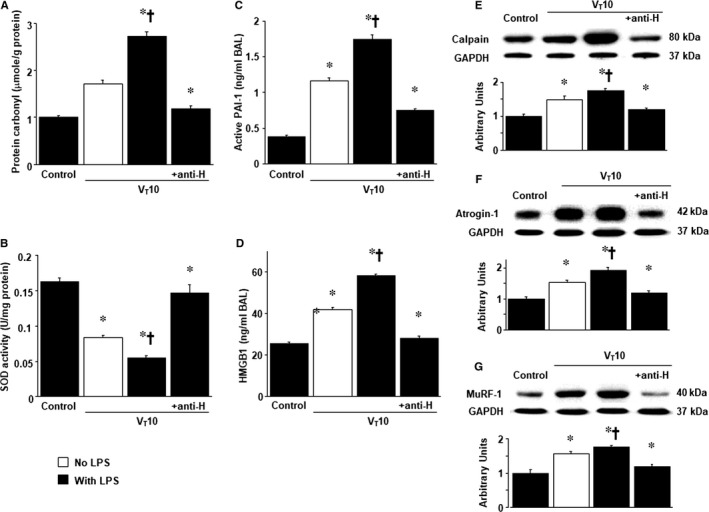
Anti‐HMGB1 antibody suppressed endotoxin‐exacerbated mechanical ventilation‐mediated oxidative stress and inflammatory cytokines production. (A) protein carbonyl groups (diaphragm), (B) SOD (diaphragm), (C) BAL fluid active PAI‐1 and (D) BAL fluid HMGB1 were from the non‐ventilated control mice and mice ventilated at a tidal volume of 10 mL/kg for 8 h with or without LPS administration (n = 5 per group). Western blots were performed using antibodies that recognize calpain (E), atrogin‐1 (F), MuRF‐1 (G) and GAPDH expression from the diaphragms of non‐ventilated control mice and mice ventilated at a tidal volume of 10 mL/kg for 8 h with or without LPS administration (n = 5 per group). Arbitrary units were expressed as relative calpain, atrogin‐1 and MuRF‐1 activation (n = 5 per group). Anti‐HMGB1 antibody, 100 mg/kg, was administered intravenously 30 min before the start of ventilation. *P < 0.05 vs the non‐ventilated control mice with LPS treatment; ^†^ *P* < 0.05 vs all other groups

**Figure 5:**
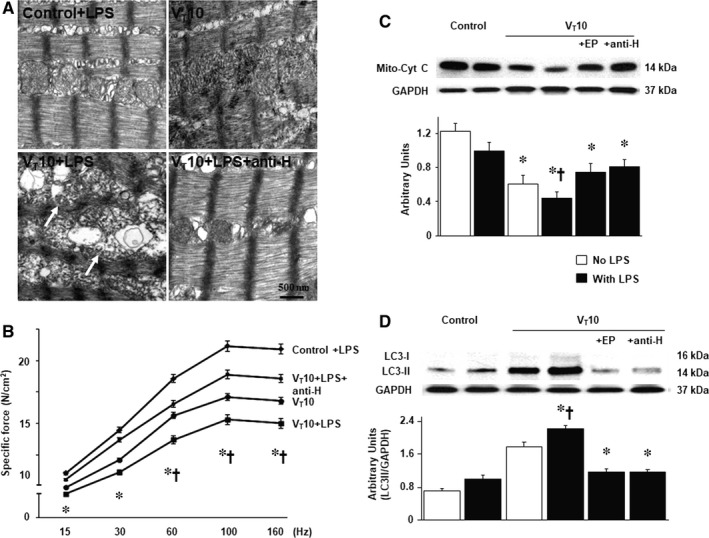
Inhibition of endotoxin‐augmented mechanical ventilation‐induced diaphragmatic injury by ethyl pyruvate and anti‐HMGB1 antibody. (A) Representative micrographs of the longitudinal sections of diaphragm (×40 000) were from the diaphragms of non‐ventilated control mice and mice ventilated at a tidal volume of 10 mL/kg for 8 h with or without LPS administration (n = 3 per group). Mitochondrial swelling with coexisting vacuole formation, loss of cristae and autophagosomes containing heterogeneous cargo is identified by arrows. (B) Diaphragm muscle‐specific force production was measured as described in Methods. (C and D) Western blots were performed using antibodies that recognize mitochondrial cytochrome C, LC3‐II and GAPDH expression from the diaphragms of non‐ventilated control mice and mice ventilated at a tidal volume of 10 mL/kg for 8 h with or without LPS administration (n = 5 per group). Arbitrary units were expressed as relative mitochondrial cytochrome C and LC3‐II activation (n = 5 per group). Ethyl pyruvate, 100 mg/kg, was given intraperitoneally 30 min before mechanical ventilation and 4 h after mechanical ventilation. Anti‐HMGB1 antibody, 100 mg/kg, was administered intravenously 30 min before the start of ventilation. *P < 0.05 vs the non‐ventilated control mice with LPS treatment; ^†^ *P* < 0.05 vs all other groups. Scale bars represent 500 nm. LC3‐II, light chain 3‐II; Mito‐Cyt C, mitochondrial cytochrome C

To determine the role of HMGB1 activation in stretch‐induced diaphragm injury, anti‐HMGB1 antibody was employed to examine whether the improvements in diaphragm abnormalities caused by the administration of ethyl pyruvate was induced through HMGB1 expression. The effects of MV (elevation in oxidative stress; active PAI‐1 and HMGB1 generation; expression levels of calpain, atrogin‐1, and MuRF‐1; diaphragm myofibrillar structures and contractilities as well as autophagosomes) in mice with endotoxaemia subjected to V_T_ 10 mL/kg were substantially reduced by the use of an anti‐HMGB1 antibody (*P* < 0.05; Figures 4 and 5A,B). Furthermore, Western blots were conducted to assess the effects of MV on endotoxin‐augmented mitochondrial damage and ALS associated with VIDD. Down‐regulated mitochondrial cytochrome C, a marker of mitochondrial structural integrity,^9^ and up‐regulated LC3‐II expression, an indicator of autophagy, 24 were observed in mice with endotoxaemia subjected to MV, but not in those without endotoxaemia subjected to MV and the non‐ventilated control mice (Figure 5C,D). However, amelioration of these injuries was observed after the administration of ethyl pyruvate or anti‐HMGB1 antibody (*P* < 0.05; Figure 5C,D). Taken together, more extensive diaphragmatic damage in mice with endotoxaemia receiving MV compared to those without endotoxaemia subjected to V_T_ 10 mL/kg (Figures 4 and 5) indicated the synergistic effects of LPS treatment.

### 3.5 Suppression of the effects of MV on endotoxin‐enhanced expression of caspase‐3 and diaphragm apoptosis by ethyl pyruvate and anti‐HMGB1 antibody

**Figure 6:**
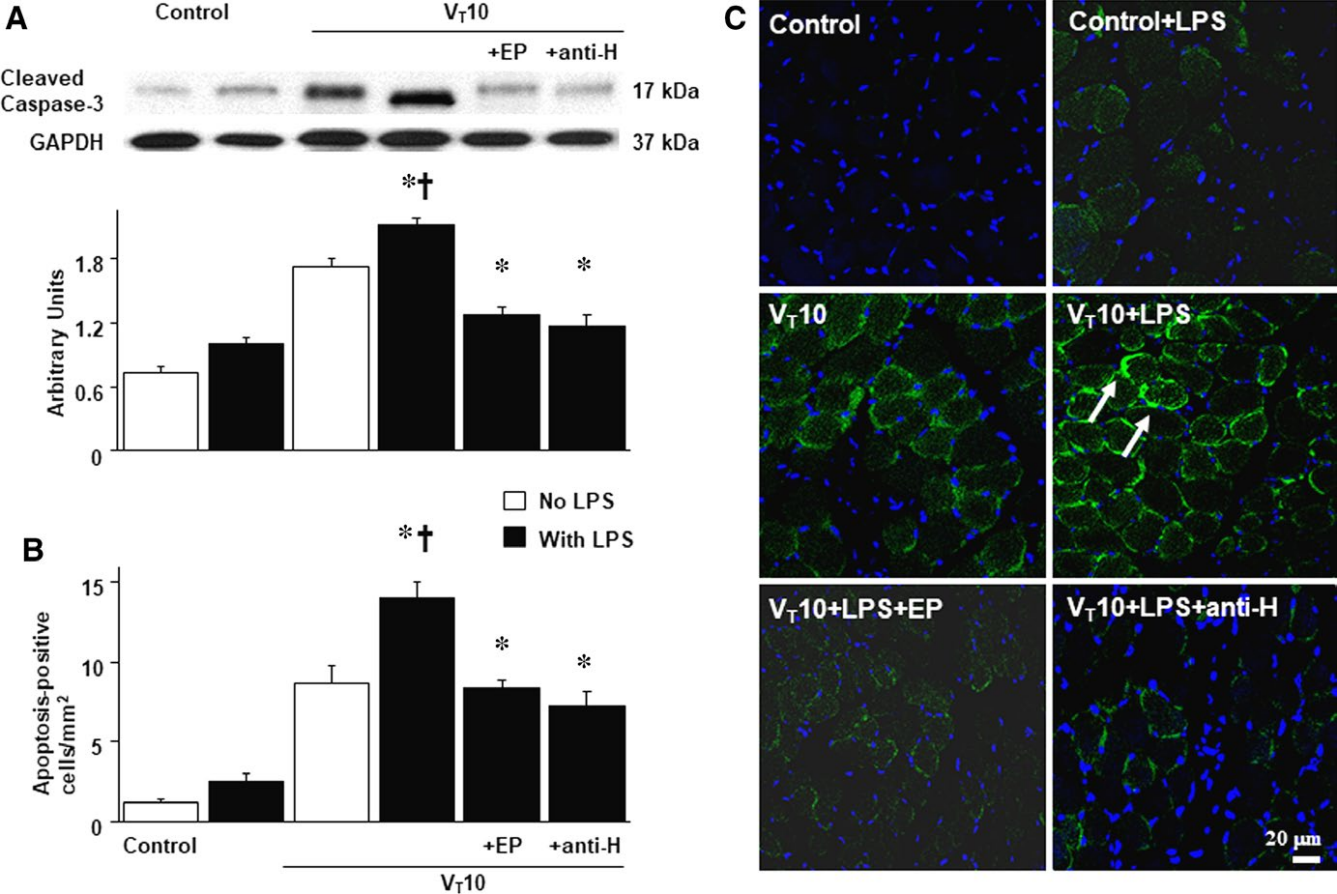
Ethyl pyruvate and anti‐HMGB1 antibody ameliorated endotoxin‐enhanced mechanical ventilation‐mediated caspase‐3 expression and apoptosis in the diaphragm. (A) Western blots were conducted using antibodies that recognize caspase‐3 and GAPDH expression from the diaphragms of non‐ventilated control mice and mice ventilated at a tidal volume of 10 mL/kg for 8 h with or without LPS administration (n = 5 per group). Arbitrary units were expressed as the ratio of cleaved caspase‐3 to GAPDH (n = 5 per group). (B and C) Representative micrographs (×400) with TUNEL staining of paraffin diaphragm sections and quantification were from the diaphragms of non‐ventilated control mice and mice ventilated at a tidal volume 10 mL/kg for 8 h with or without LPS administration (n = 5 per group). Ethyl pyruvate, 100 mg/kg, was given intraperitoneally 30 min before mechanical ventilation and 4 h after mechanical ventilation. Anti‐HMGB1 antibody, 100 mg/kg, was administered intravenously 30 min before the start of ventilation. Apoptotic cells are identified by arrows. A bright green signal indicates positive staining of apoptotic cells, and shades of dull green signify non‐reactive cells. *P < 0.05 vs the non‐ventilated control mice with room air; ^†^ *P* < 0.05 vs all other groups. Scale bars represent 20 μm. TUNEL, terminal deoxynucleotidyl transferase‐mediated dUTP‐biotin nick end‐labelling

**Figure 7:**
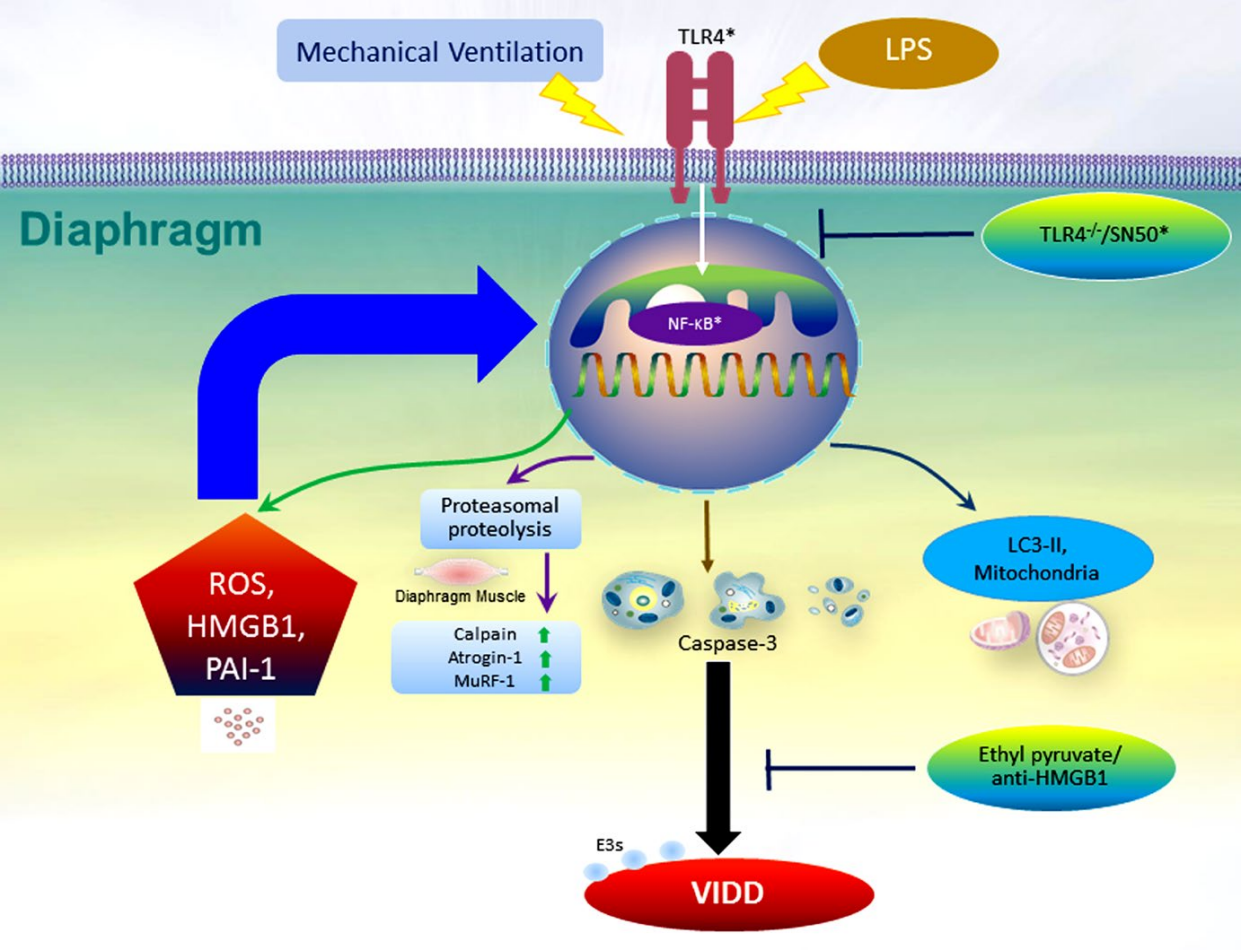
Schematic figure illustrating the signalling pathway activation with mechanical ventilation and endotoxaemia. Endotoxin‐mediated augmentation of mechanical stretch‐induced inflammatory cytokine production and diaphragm injury was alleviated after the administration of ethyl pyruvate and anti‐HMGB1 antibody.^31^ HMGB1, high‐mobility group box‐1; LC3‐II, light chain 3‐II; LPS, lipopolysaccharide; MuRF‐1, muscle ring finger‐1; NF‐κB, nuclear factor κappa B; PAI‐1, plasminogen activator inhibitor‐1; ROS, reactive oxygen species; TLR4, toll‐like receptor 4; VIDD, ventilator‐induced diaphragm dysfunction. *Reference 31

Studies have demonstrated that caspase‐3 is crucial to the intrinsic apoptotic pathway.^20^, ^31^ Capase‐3 expression and TUNEL staining were used to identify the roles of the caspase‐3 pathway and apoptosis of diaphragm myofibrils in endotoxin‐related VIDD (Figure 6). A substantial up‐regulation in caspase‐3 expression and the emergence of TUNEL‐positive apoptotic nuclei in the murine diaphragm occurred in mice with endotoxaemia subjected to V_T_ 10 mL/kg, but not in those without endotoxaemia subjected to V_T_ 10 mL/kg and the non‐ventilated control mice (Figure 6B,C). Specifically, the activation in caspase‐3, as well as MV‐ and endotoxin‐enhanced apoptosis, in the murine diaphragm was restored following the administration of ethyl pyruvate as well as anti‐HMGB1 antibody. Collectively, our results suggest that endotoxin‐ and concurrent MV‐mediated oxidative loads and the inflammatory reactions in the diaphragm were protected by the inhibition of the HMGB1 pathway (Figure 7).

## 4 DISCUSSION

In clinical practice, MV is required to provide adequate ventilation and oxygenation in patients with sepsis and respiratory insufficiency. Infection can trigger substantial diaphragm dysfunction in animal and clinical human studies of sepsis.^7^, ^15^, ^26^ However, the use of MV can lead to the rapid development of diaphragm weakness and ventilator‐induced lung injury (VILI) in humans.^3^, ^4^ Recently, MV was determined to likely aggravate diaphragm injuries in a murine model of endotoxaemia.^31^ The combination of infection and ventilator‐induced diaphragm disuse exacerbated the diaphragmatic force‐generating contractility in mechanically ventilated patients.^5^ Diaphragm dysfunction is a critical determinant that negatively influences the clinical outcome of critically ill patients and typically accompanies prolonged MV exposure, and increases ventilator‐associated complications, and mortality.^1^ Thus, potent pharmacological agents should be sought to effectively ameliorate the diaphragm dysfunction induced in such critical situations and facilitate the successful weaning of MV. In this study, we demonstrated that ethyl pyruvate can (a) mitigate oxidative stress and improve antioxidant ability, (b) decrease inflammatory cytokines HMGB1 and PAI‐1, (c) ameliorate muscle proteolysis and apoptosis, (d) attenuate mitochondrial injury and autophagy and (e) restore the ultrastructural integrity and muscle contractility in a mouse model of VIDD with endotoxaemia. Moreover, we investigated the deleterious role of HMGB1 in mediation of the pathogenic mechanisms of diaphragm injury.

HMGB1, a non‐histone chromosomal protein, is translocated to the cytosol from the nucleus and then released from cells under stressful situations. Thus, HMGB1 acts as a catastrophic inflammatory cytokine passively released by damaged tissues or actively secreted from activated inflammatory cells during sepsis.^26^, ^40^ Extracellular HMGB1 can be expressed as a DAMP by binding to TLR4, triggering inflammatory responses for host defence during early sepsis and producing cytokine cascades in later stages of sepsis.^40^ Furthermore, previous studies of ALI in mice and monocytes pretreated with HMGB1 demonstrated that HMGB1 triggered lung inflammation with neutrophil sequestration and releases of pro‐inflammatory cytokines, including IL‐1β, macrophage inflammatory protein‐2 and tumour necrosis factor‐α.^41^, ^42^ Elevated HMGB1 levels have been observed in the pulmonary epithelial lining fluid of septic patients.^43^ Moreover, HMGB1 was revealed to accelerate muscle fatigue, and is believed to be an early trigger of skeletal muscle dysfunction through binding to TLR4. HMGB1 is also believed to impair sarcoplasmic reticulum Ca^2+^ release in patients with myositis.^30^ High HMGB1 concentrations were observed to be associated with patients who required long‐term MV use and those with VAP.^44^ Previous studies on VILI have determined that MV can up‐regulate the production of HMGB1 and that HMGB1 is a pivotal mediator in the development of VILI.^29^, ^45^ Furthermore, a moderate V_T_ of MV can aggravate LPS‐induced lung injury by up‐regulating HMGB1.^46^ However, no studies have investigated the role of HMGB1 in VIDD and endotoxaemia, a critical situation simulating the clinical scenario. Previous related studies have demonstrated that HMGB1 expression in the diaphragm is associated with impaired diaphragm contractility and diaphragm dysfunction in septic animals with peritonitis.^15^, ^26^, ^28^ Our present study demonstrated that a moderate V_T_ of ventilation can enhance the HMGB1 production from the diaphragm of LPS‐challenged mice with endotoxaemia and worsen diaphragmatic injury, as evident in increased oxidative stress, inflammation, atrophy, apoptosis, mitochondrial injury and autophagy. These deleterious effects were attenuated by using the anti‐HMGB1 antibody. Although ROS and early cytokines are important to contribute to VIDD, and herein, we provide the evidence that using anti‐HMGB1 antibody can elevate the SOD activity and suppress the oxidants, inflammatory cytokines and VIDD comparable to the beneficial effects of ethyl pyruvate in this model (Figures 3, 4, 5, 6). In addition, we proved that MV induces diaphragmatic injury in a mouse model of endotoxaemia through activation of the TLR4/NF‐κB signalling pathway.^31^ This inspired us to investigate the role of HMGB1, a pivotal ligand of the TLR4 receptor, and develop an effective agent to improve VIDD during endotoxaemia.

Ethyl pyruvate, a simple aliphatic ester, is derived from the endogenous metabolite of pyruvic acid. Ethyl pyruvate can function as both a potent ROS scavenger and anti‐inflammatory agent through the up‐regulation of haem oxygenase‐1 or suppression of the NF‐κB and p38 mitogen‐activated protein kinase (MAPK)‐mediated inflammatory responses.^47^, ^48^, ^49^ Ethyl pyruvate can also act as an HMGB1 inhibitor and ameliorate the systemic inflammation and organ dysfunction in animal models of endotoxaemia and sepsis.^33^, ^50^ In addition, a proprietary formulation of ethyl pyruvate was proven to be safe and well tolerated at clinically relevant dosages in clinical trials on healthy subjects and patients who underwent cardiac surgery.^47^ However, the application of ethyl pyruvate for diaphragmatic injury induced by the combination of MV and LPS has not been investigated. To the best of our knowledge, this is the first study to examine the salutary effects of ethyl pyruvate on diaphragmatic dysfunction associated with inflammation, proteolysis, apoptosis, autophagy and mitochondrial injury. A considerable amount of research has documented that oxidative stress mediates the release of the redox‐sensitive protein HMGB1 during sepsis and can trigger mitochondrial permeability transition, inducing apoptosis, autophagy and mitochondrial damage by integrating the HMGB1.^51^ Ethyl pyruvate is protective in the setting of experimental sepsis partly by inhibiting HMGB1 release and accumulation.^52^ Mechanistically, ethyl pyruvate was determined to promote the deacetylation of HMGB1 through the signal transducer and activator of transcription 1 (STAT1) phosphorylation up‐regulating sirtuin1, thereby reducing the release of HMGB1 in LPS‐activated murine macrophages.^53^ Li et al also documented that ethyl pyruvate preserved the mitochondrial integrity by inhibiting the nucleotide‐binding domain, the leucine‐rich–containing family and the pyrin domain‐containing‐3 (NLRP3) agonist‐induced mitochondrial damage by acting as an NLRP3 inflammasome inhibitor in human acute monocytic leukaemia cell lines.^54^ In the present study, we demonstrated that ethyl pyruvate can ameliorate diaphragmatic injury through the reduction of oxidative stress, inflammation, proteolysis, apoptosis, autophagy and mitochondrial injury, all of which are achieved through the inhibition of HMGB1. Although Su et al showed that a single dose of ethyl pyruvate (100 mg/kg) decreased NF‐κB and plasma cytokines in 3 h, but those inflammatory parameters were elevated in 9 hours in high‐dose LPS (30 mg/kg)‐challenged mice for survival test.^55^ In the subsequent study, ethyl pyruvate at different doses of 100 mg/kg or 50 mg/kg was proven to reduce the mortality from endotoxin‐induced ALI and the permeability index in mice.^34^ Notably, the beneficial effects of 100 mg/kg ethyl pyruvate were superior compared to those of 50 mg/kg, which are consistent with our dose‐response results (Figure S1). A single intraperitoneal dose of ethyl pyruvate was able to reduce methaemoglobinaemia induced by dapsone for a short period of time of 3‐6 h.^56^ Thus, a second dose was designed to be given after the mice subjected to 4 hours of MV at our protocol. As for the toxicity of ethyl pyruvate, dosage up to 150 mg/kg was proven to be a safe and effective medication in endotoxaemic horses, and ethyl pyruvate (90 mg/kg) at 6‐hour intervals for five more doses was used with safety in human trials.^57^, ^58^ In addition, anti‐HMGB1 antibody (100 mg/kg) can significant attenuate diaphragm dysfunction in septic rats, and the beneficial effect was comparable to the highest dose of 250 mg/kg, but was superior compared to the dose of 25 mg/kg, which are in accordance with our results (Figure S1).^28^

The present study had some limitations. First, in activated neutrophils, HMGB1 induced p38 MAPK, extracellular signal‐regulated kinases 1/2 and serine/threonine kinase/protein kinase B. It also increased the production of NF‐κB–dependent inflammatory cytokines, including tumour necrosis factor‐α, IL‐6 and macrophage inflammatory protein‐2, in lung tissue during infection and injury.^37^, ^42^ Evidence has indicated that acetylation of HMGB1 prevents its entry into the nucleus and causes the release of HMGB1 from cells, thus initiating inflammation, as well as LPS‐activated acetylation of HMGB1, through Janus Kinase/STAT signalling in TLR4‐activated macrophages.^27^, ^59^ Further investigation is necessary to explore other possible molecular mechanisms that involve HMGB1 and the advantageous effects of ethyl pyruvate in preventing patients with endotoxaemia from developing VIDD. Second, we utilized the anti‐HMGB1 antibody in the study since HMGB1‐/‐ mice die shortly after birth due to a defect in the transcriptional activation of the glucocorticoid receptor.^29^ However, Kim et al reported that intranasal delivery of HMGB1 siRNA provides the neuroprotection in the ischaemic brain mediated by target gene knockdown.^60^ Further alternative agents are expected to be researched in future studies.

By using an in vivo murine model of VIDD with endotoxaemia, we demonstrated that MV worsened LPS‐challenged diaphragm injury and dysfunction, as reflected in increased oxidative stress and higher levels of inflammatory cytokines HMGB1 and PAI‐1; proteolytic proteins calpain, atrogin‐1 and MuRF‐1; apoptotic enzyme caspase‐3; autophagic marker LC3‐II expression; and more mitochondrial injury. This was also demonstrated through reduced antioxidant activity, mitochondrial cytochrome C levels and muscular force‐generating capacity. The deleterious effects of MV on the diaphragm of mice with endotoxaemia can be alleviated by pharmacological inhibition with ethyl pyruvate, a HMGB1 inhibitor, or the use of anti‐HMGB1 antibody, which block the HMGB1‐mediated diaphragmatic injury and restore the ultrastructural integrity and functional contractility of the diaphragm. Understanding the beneficial mechanisms of ethyl pyruvate achieved through the regulation of HMGB1 may contribute to the development of novel biomarkers for monitoring diaphragmatic dysfunction during the use of MV and contribute to the growing knowledge of precise pathogenic mechanisms of combinatorial infection and MV involved in the development of diaphragmatic damage.

## CONFLICT OF INTEREST

The authors confirm that there are no conflicts of interest.

## AUTHOR'S CONTRIBUTION

LFL and YYL performed the experiments and wrote the manuscript. NHC, CHC, SWL, KCK, HCH and GJC designed the experiments and analysed the data.
