# Supplementary figures and images for: Ethyl pyruvate attenuates ventilation‐induced diaphragm dysfunction through high‐mobility group box‐1 in a murine endotoxaemia model
Source: J Cell Mol Med. 2019 Jun 10;23(8):5679–91. doi: 10.1111/jcmm.14478 (PMC6652995; doi:10.1111/jcmm.14478)

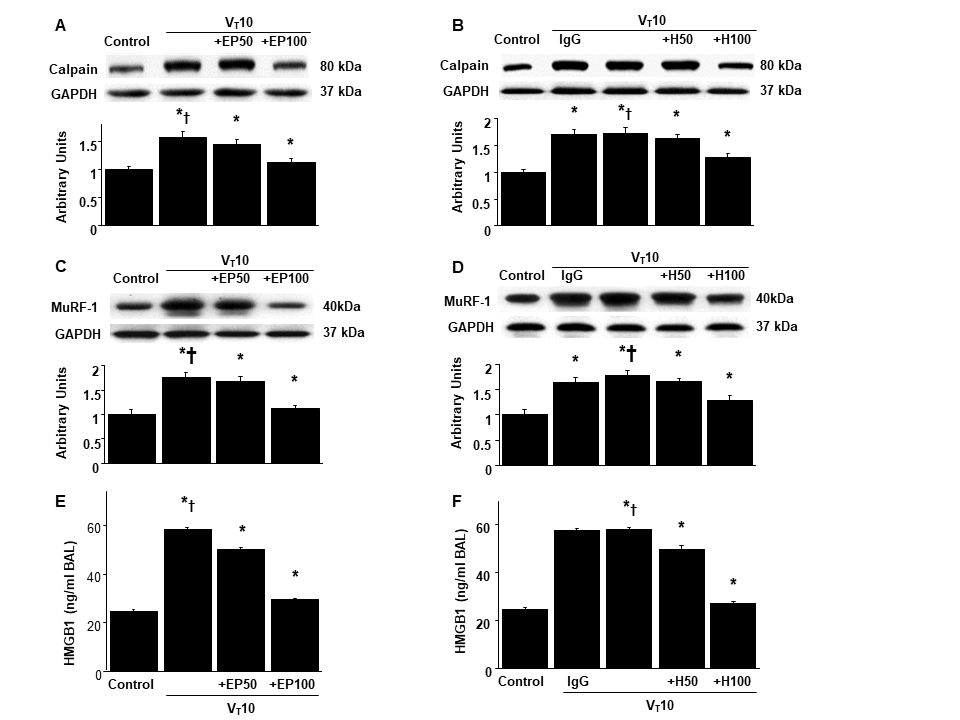

Supplement: Supplementary file 1 [file JCMM-23-5679-s001.tif]
