# Supplementary material for: Ethyl pyruvate attenuates ventilation‐induced diaphragm dysfunction through high‐mobility group box‐1 in a murine endotoxaemia model
Source: J Cell Mol Med. 2019 Jun 10;23(8):5679–91. doi: 10.1111/jcmm.14478 (PMC6652995; doi:10.1111/jcmm.14478)
Supplement: Supplementary file 2 [file JCMM-23-5679-s002.doc]

**Table 1. Physiologic conditions at the beginning and end of ventilation.**

|  | Nonventilated | Nonventilated  +LPS | V_T_ 10 mL/kg | V_T_ 10 mL/kg  +LPS | V_T_ 10 mL/kg  +LPS+EP | V_T_ 10 mL/kg  +LPS+anti-H |
| --- | --- | --- | --- | --- | --- | --- |
| PH | 7.42±0.05 | 7.38±0.04 | 7.36±0.06 | 7.38±0.05 | 7.39±0.06 | 7.37±0.06 |
| PaO2 (mmHg) | 98.6±0.3 | 95.9±0.3 | 87.2±0.3* | 85.1±2.9* | 87.6±2.2* | 89.1±2.1* |
| PaCO2 (mmHg) | 39.1±0.4 | 39.4±0.3 | 37.2±1.4 | 38.2±1.6 | 37.6±1.3 | 38.4±1.5 |
| MAP (mmHg) |  |  |  |  |  |  |
| Start | 85.5±1.2 | 83.9±0.5 | 85.6±1.4 | 82.4±2.3 | 84.9±2.1 | 84.7±1.6 |
| End | 85.0±0.5 | 81.0±0.4 | 77.8±2.5* | 75.1±2.5* | 78.3±2.3* | 79.2±2.5* |
| PIP (mmHg) |  |  |  |  |  |  |
| Start |  |  | 15.7±1.2 | 16.1±1.2 | 15.9±1.3 | 15.8±1.2 |
| End |  |  | 16.9±1.7 | 17.8±1.5 | 17.2±1.4 | 17.1±1.6 |

At the end of the study period, we obtained data of mean arterial pressure and arterial blood gases from the nonventilated control mice and mice ventilated at a tidal volume of 10 mL/kg for 8 h with or without endotoxemia (n = 10 per group). The normovolemic statuses of mice were maintained by monitoring mean artery pressure. Data are presented as means ± SDs. * indicates that P < 0.05 when compared to the nonventilated control mice with endotoxemia. LPS = lipopolysaccharide; MAP = mean arterial pressure; PIP = peak inspiratory pressure; EP = ethyl pyruvate; Anti-H = anti-high-mobility group box-1 antibody; V_T_ = tidal volume.
